# Current evidence on the use of anti-RAAS agents in congenital or acquired solitary kidney

**DOI:** 10.1080/0886022X.2017.1361840

**Published:** 2017-08-14

**Authors:** Mariadelina Simeoni, Annarita Armeni, Chiara Summaria, Annamaria Cerantonio, Giorgio Fuiano

**Affiliations:** Department of Nephrology and Dialysis, Magna Graecia University of Catanzaro, Catanzaro, Italy

**Keywords:** Congenital and acquired solitary kidney, RAAS system, RAAS inhibition, nephron hyperplasia, nephron hypertrophy

## Abstract

**Rational:** The inhibition of renin–angiotensin–aldosterone system (RAAS) is a major strategy for slowing the progression of chronic kidney disease (CKD). The utility of anti-RAAS agents in patients with congenital or acquired solitary kidney is still controversial.

**Objective:** A systematic literature review was conducted.

**Main findings:** The conclusions of the few available studies on the topic are homogeneously in agreement with a long-term reno-protective activity of anti-RAAS drugs in patients with solitary kidney, especially if patients are hypertensive or proteinuric. However, angiotensin 2 (ANG2) levels permit a functional adaptation to a reduced renal mass in adults and is crucial for sustaining complete kidney development and maturation in children. A hormonal interference on ANG2 levels has been supposed in women. Consequently, at least in children and women, the use of ARBs appears more appropriate.

**Principle conclusions:** Available data on this topic are limited; however, by their overall assessment, it would appear that anti-RAAS drugs might also be reno-protective in patients with solitary kidney. The use of ARBs, especially in children and in women, seems to be more appropriate. However, more experimental data would be strictly necessary to confirm this hypothesis.

## Introduction

Solitary kidney is a congenital or acquired condition characterized by a reduced renal mass [1]. Defects in kidney development, genetically or non-genetically determined, are part of the Congenital Anomalies of the Kidney and Urinary Tract (CAKUT) [2]. The epidemiological magnitude of the kidney malformations is underestimated and often accidentally discovered only in adult age with evidence for renal function impairment [3]. Renal function substitution with dialysis is needed in a significant number of patients (40% in children and 0.6% in adults) inducing a quality of life impairment [4,5]. CAKUT is an extensive clinical issue ranging from high (complete renal agenesis) to mild (renal hypodysplasia, multicystic kidney dysplasia, ureteropelvic junction obstruction, megaureter, posterior urethral valves, and vesicoureteral reflux) severity. However, renal dysplasia and vesicoureteric reflux are the most frequent causes of end-stage renal disease in childhood. Dysplastic kidney might present also hypoplastic and shows a significant nephron deficit appearing undifferentiated or metaplastic. Patients with renal dysplasia and hypoplasia are prone to urinary tract infections or chronic renal failure development. The retrograde flow of urine from the bladder into the ureter and toward the kidney is due to a dysfunctional vesicoureteric junction and is called vesicoureteral reflux. This condition is often complicated with urinary infections, renal scarring, hypertension and end-stage renal disease in both children and young adults [6].

Acquired solitary kidney depends on kidney surgical removal due to several different causes (cancer, trauma, receiving kidney transplant, living kidney donation, post-biopsy kidney hemorrhage, etc.) [3]. After nephrectomy, remnant kidney undergoes a series of compensatory changes and Doppler ultrasonography should be periodically applied in the follow up. In fact, assessment of resistive index (RI) and pulsatily index (PI) provide important information on adaption ability of the remnant kidney and might reveal early signs of organ failure [7].

An acute reduction of the renal mass has a variable impact on the renal function, compared with congenital renal malformations [8]. It is possible that different compensation mechanisms are involved in the two conditions.

Renin–angiotensin–aldosterone system (RAAS) inhibition is a major therapeutic approach for slowing chronic kidney disease (CKD) progression [9,10]. Proteinuria occurrence is a frequent complication in patients with a single functioning kidney, representing an aggravating cause of GFR decline. In a study by Liern et al. [11] antiproteinuric efficacy of enalapril was reported and found synergic with a normal dietary protein intake.

Whether the use of anti-RAAS agents exerts its known nephro-protective activity also in patients with renal malformations or acquired solitary kidney is a debated field of investigation in childhood and an under-investigated topic in adult age. We aimed at systematically reviewing current literature for the assessment of the prevalent evidence on the effectiveness and safety of anti-RAAS agents use in all age patients with kidney development anomalies.

## Compensation mechanisms in congenital and acquired solitary kidney

A renal compensation in both congenital and acquired solitary functioning kidney is evident with high clinical variability [12]. Nevertheless, it is uncertain if being born with a single kidney or having lost one kidney during the life would have a different impact on renal outcomes. Mechanisms able to compensate renal mass loss or kidney under-development have been described in both cases and mainly in studies conducted on animals [13]. Congenital single functioning kidney, compared with acquired solitary kidney, might be less hypertrophic compared to acquired solitary kidney. A comparative analysis is desirable.

Renal malformations occur in 0.3–1.6/1000 newborns [14,15] and represent approximately 30% of all prenatally diagnosed malformations [16]. Their impact on renal function decline is variable and depends on kidney size and dysplasia grading at birth. As consequence, a congenital renal disorder can be found in almost 60% of children with CKD showing a rapid progression to ESRD only in severe newborns [17]. In milder cases, instead, renal function tends to improve during the first 3–4 years of life and remains stable up to the adolescence, when a new decline can occur and progress to ESRD in approximately 25% of patients. Conversely, in adult age, renal malformations are accidentally diagnosed and rarely lead to ESRD. However, a common association is established with hypertension and cardiovascular complications in adult patients with congenital renal malformations [18].

Clinical variability in patients with congenital solitary kidney appears to depend on the severity of the kidney disorder and may be linked to the gestation age at which the insult is installed. The variable clinical response to congenital renal malformations has to be searched in compensatory fetal renal growth mechanisms.

Compensatory mechanisms in congenital renal malformations have been investigated in animals with difficult translation of the data to humans because renal ontogeny is species specific [19,20]. However, the ovine kidney development process is very similar to that of human kidney.

A model of mono-nephrectomized fetal sheep has been reproduced and compared with sham operated ovine fetuses to investigate functional and morphological responses to a partial renal loss at 100 d of gestation, when nephrogenesis is still very active [21]. One month after surgery, nephrectomized ovine fetuses developed marked compensatory hyperplasia with a significantly increased number of glomeruli showing mild hypertrophy. Furthermore, in nephrectomized ovine fetuses, renal function was found to be similar to that of sham operated ovine fetuses. However, in 6 months old uninephrectomized male sheep, glomerular filtration rate (GFR) and renal blood flow (RBF) were found to be reduced, while blood pressure increased. An up-regulation of RAAS system was hypothesized but not confirmed by either molecular biology or immunostaining techniques.

**Figure 1.**
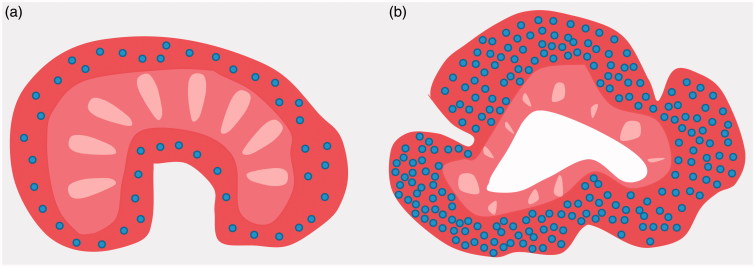
Glomerular distribution and conformation of a normal (a) and contralateral to nephrectomized fetal kidney (b) during active nephrogenesis stage.

**Figure 2.**
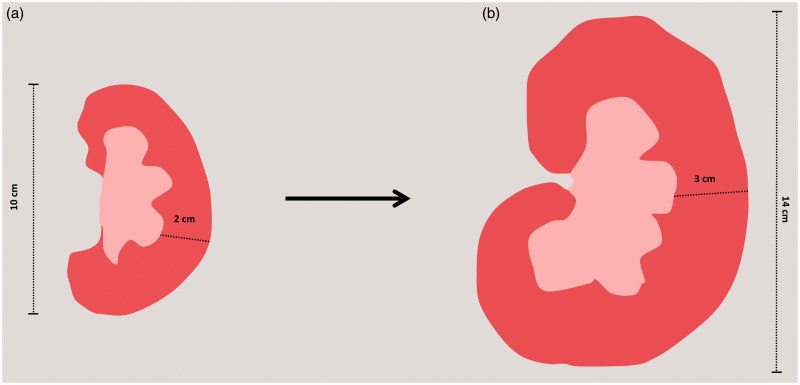
Alteration in both size and cortical thickness in a normal (c) and in a solitary single kidney (d) in renal sections.

In addition, the development of pig kidney is comparable with humans. Congenital functioning solitary kidneys in 26 week-old pigs have been anatomically compared with normal controls by van Vuuren et al. Both kidney weight and nephron number were found to be significantly increased in congenital solitary kidney [19]. A comparison of histological patterns was also conducted showing that glomerular and tubular size in congenital solitary kidneys is similar to controls, while the number of nephrons was found to be doubled. The total mass of glomeruli as a percentage of cortical mass was considerably greater in the congenitally solitary kidneys than in controls [19] (Figures 1 and 2).

In a case report, Maluf described a comparison between a congenital solitary kidney dissected from the cadaver of a murdered young man and one of the two normal kidneys from the cadaver of another healthy young man. Both anatomical and histological analysis were performed, showing that congenital solitary kidney was normal in shape, position, and size but significantly heavier with a significantly thicker renal cortex than in the normal control. The histological study evidenced the presence of a hyperplasic pattern, as documented in the previous studies conducted on animals [22].

Based on the ‘hyperplasia hypothesis’, the adaptive response to congenital nephron deficiency is possible at an early fetal age, when the number of glomeruli, due to the active mitosis, can still be increased [23].

Any renal mass loss occurring after mitosis process inactivation in fetal life would lead to a different compensation mechanism. On this basis, congenital solitary kidney might share a similar clinical outcome with acquired solitary kidney when a tardive insult to normal kidney development occurs. This might be the explanation for an increased renal and cardiovascular risk in individuals born with a reduced renal mass [18,24].

Acquired solitary kidney consists of an acute surgical renal mass loss necessary in several conditions: donation for transplant, cancer, trauma, renal bleeding, and renal infectious complications [8].

An increase of glomerular flow in the remnant kidney after mono-nephrectomy occurs and involves a series of compensatory changes inducing glomerular hypertrophy appearance. In this frame, hypertrophy is generally considered advantageous as expression of an adaptive response of the remnant kidney to a sudden renal mass loss and results in an increase of single nephron glomerular filtration rate within 1 month after surgery [25].

Of note, uninephrectomy experiments in rats and humans have shown an inverse correlation between age and GFR increase, indicating impaired adaptation in the long term [26].

Sudden massive loss of nephrons compensated by glomerular hypertrophy may represents a pre-pathological condition culminating in glomerulosclerosis and progressive renal tissue damage. In an epidemiological American study, 96,217 living kidney donors having a normal baseline GFR were monitored and compared with 20,024 healthy controls and hypertension, CKD and ESRD were significantly prevalent in living kidney donors [27].

Similar results were reported by Mjøen et al. in study based on 24.9 years follow-up and conducted on 1901 Norwegian living kidney donors and 32,621 healthy subjects included in the Health Study of Nord Trøndelag (HUNT). However, Mjøen supposed that the higher incidence of ESRD observed in living kidney donors could depend on hereditary factors since the risk of ESRD in biologically unrelated donors was smaller [28].

## Tubular adaption to renal mass reduction

Tubular adaption to renal mass reduction has been evaluated only in few studies and mainly in animal models. However, it is evident that in congenital and acquired single kidney renal tubule structure and function is modified similarly to glomerulus. In order to provide a proportional increase of tubular activity in response to compensatory glomerular hyperfiltration, tubular part of the nephron is enlarged and all ionic and acid-base transport functionalities are enhanced [1,29]. In a micropuncture study conducted on 2–4 weeks old rats, Hayslett et al. [30] compared the tubular adaption in nephrectomized animals to normal controls and found a prevalent proximal tubular volume increase in tubular volume only in the nephrectomized group. Fluid transit time through visible distal tubular segments was also measured. In nephrectomized rats, it was significantly prolonged suggesting a glomerular hyperfiltration-induced disproportion in Henle’s loop pars recta elongation. Authors also found an increase in active transport of sodium by tubular cells suggesting an over-activity of sodium pump. In conclusion, water and sodium tubular permeability appeared deeply modified in response to compensatory hypertrophy.

Gadalean et al. have observed similar functional and morphological tubular adaption mechanisms in patients with both congenital and acquired single kidney. However, in patients with congenital renal mass reduction, adaptive tubular changes were earlier, suggesting a congenital adaption to renal anomalies, presumably with better renal outcomes [31]. Conversely, in patients with acquired single kidney, a similar adaption grading to congenital single kidney occurs only within several years and might be associated with a faster decline of renal function. Bohle et al. demonstrated that tubular adaption is followed by tubular injury onset. In a cross-sectional study, in fact they found high urinary levels of *N*-acetyl-beta-d-glucosaminidase (NAG), alpha 1-microglobulin and albumin in patients with congenital and acquired single kidney. Of note, patients affected by acquired single kidney showed even higher levels of urinary tubular biomarkers levels compared to patient with congenital single kidney [32].

## RAAS expression and activity in congenital and acquired solitary kidney

RAAS regulates renal vasomotor activity, maintains optimal salt and water homeostasis, and controls kidney development in the fetal life [33]. Moreover, this important system is implicated in the progression of renal damage with recent evidence for a pleiotropic activity of RAAS components, able to induce hyper-expression of several damaging cytokines and growth factors [9,10,34].

Consequently, this important system plays a central role in the maintenance of the renal function in patients with a solitary kidney and its grade of expression could influence the renal damage progression, as fully described in the literature [35–39].

Initial studies were conducted on animals in which a remnant kidney model was reproduced in rodents undergone partial or total uni-nephrectomy in which a hyper-activation of the RAAS system has been demonstrated as an expression of the main mechanism of compensation to renal mass loss [40]. Several comparative experiences have also been reported in humans with evidence for a higher risk to develop CKD, hypertension and proteinuria in patients having one surgically ablated kidney in comparison with patients with congenital solitary kidney [8].

Renal artery stenosis is associated with a functional kidney exclusion and CKD development. This condition is somehow comparable to solitary acquired kidney. An important similarity consists in the overactivity of RAAS demonstrated in both animal models and humans with ischemic nephropathy due to renal artery stenosis. In this condition, in fact, blood pressure control should include the use of anti-RAAS agents [41].

A possible difference in RAAS expression and activity has to be supposed in solitary and acquired kidney, although has not yet been demonstrated. Hypertrophic glomeruli have an increased glomerular flow sustained by angiotensin 2 (ANG2)-induced efferent arteriolar vasoconstriction [42]. On the contrary, it is conceivable that in hyperplastic congenital solitary kidney the single-nephron GFR might be normal in absence of RAAS hyperactivation [42].

All RAAS components play a crucial role in the metanephric stage of kidney development and are essential for systemic blood pressure and renal blood flow maintenance in fetal life [33]. A role in the acid base regulation could also be hypothetized [43].

More specifically, ANG2 is fundamental in branching morphogenesis, vasculogenesis, development of the papilla and renal concentrating mechanisms, as reported in gene targeting studies conducted on animals [16,44,45].

This evidence appears translatable to humans in consideration of the known teratogenic effects of anti-RAAS agents used in the pregnancy [46]. Several interaction pathways between RAAS activity and tissue expression and estrogen are reported in the literature involving oxidative stress and vasoconstriction mechanisms. Women appear to have a lower RAAS activity especially in term of ANG2 production and AT1R tissue expression providing a lower susceptibility to develop hypertension and cardiovascular complications. These interactions are also differently modulated during the life in relation to different ovarian stages. However, in the case of renal mass loss, a deficiency of ANG2 might be associated with a worsen remnant kidney adaption [47].

Moreover, the influence on renal development process by environmental or genetic defects induces such a variable clinical presentation of congenital single kidney, for which a different degree of compensatory hypertrophy or hyperplasia is possible. Undoubtedly, a different extent of RAAS expression could be an explanation for this difference. However, also an impaired interaction of ANG2 and its receptor (AT1R) has been reported to influence cellular division and hyperplasia degree in CAKUT children [46].

Accordingly, in an editorial comment by Corbani et al. [48] is reported that patients with congenital solitary kidney should not be considered as an homogeneous category and patients showing an early appearance of hypertension, proteinuria and GFR decline might have, therefore, a prevalence of glomerular hypertrophy within their compensatory pattern [49].

## Are anti-RAAS agents advantageous in patients with solitary kidney? – methods for literature review

Not many studies investigated an eventual positive effect of anti-RAAS agents use in patients with congenital or acquired solitary kidney, thus this therapeutic approach remains controversial. We searched the available literature data focusing on the impact of drugs interfering with RAAS system on the function of the solitary kidney. Studies examining this topic were detected by a computerized research of all English-language articles in the electronic database PubMed. We carried out a systematic search of full-text papers, published between 2006 and 2015 by combining the following Medical Subject Heading (MeSH) terms: ‘Renoprotection in single kidney’, or ‘ACE-inhibitors and renal ablation’, or ‘Fetal uninephrectomy and Renin Angiotensin System inhibition’, or ‘Renal damage frequency in patients with single kidney and anti-RAAS agents’ or ‘Renal mass reduction and ARBs and ACEi and direct renin inhibition’. Four hundred and ninety-four references were initially retrieved. Three hundred and fifty references were excluded because not pertinent with our topic. One hundred and thirty-nine papers were discharged after full text analysis for the following reasons: 50 papers did not add new information, 69 papers were narrative reviews, and 20 papers were reported in other than English languages. Ten articles were finally included in our review.

## Evaluating the impact of anti-RAAS drugs on the function of congenital or acquired solitary kidney

Renal function decline rate represents a major issue in patients with congenital or acquired solitary kidney. The adaption to renal mass loss is variable and mainly sustained by RAAS modulation, especially in patients with a prevalent hypertrophic compensation. The few available studies testing anti-RAAS agents impact on the function of solitary kidneys were conducted mainly in animal models and only limited data were reported in humans. The spectrum of drugs interfering with RAAS is wide and different pharmacodynamics and pharmacokinetics are available to achieve renoprotection; however, side effects often represent the major limitation to RAAS inhibition (Table 1). Available data were mostly derived on ACEi and ARBs use in solitary kidney models, while in none of the included studies the direct inhibition of renin was tested. Considering the relevant pleiotropic effects of the blockade of pro-renin–renin–MAP kinase axis [34], treatment of solitary kidney patients with a direct renin inhibitor, such as aliskiren, deserves future investigation.

**Table 1.** Describes pharmacological characteristics of the three classes of anti-RAAS agents: ACE inhibitors (ACEi), direct renin inhibitors and angiotensin receptor blockers (ARBs). Based on their different mechanism of action, these classes of drugs are used for different treatment, even if the risk of main adverse effects is similar for all the categories.

Classes of drugs	Mechanism of action	Indications	Half-life	Bradichinine accumulation	Impact on plasma renin concentration and activity	Main adverse effects
Angiotensin converting enzyme inhibitors (ACEi)	RAAS down-regulation by inhibition of the ACE enzyme activity, which converts Angiotensin I in Angiotensin II	Treatment of hypertension, heart failure, chronic non-diabetic and diabetic renal disease, dry cough	2–40 h	Yes	↑ Renin concentration ↔ Renin activity	Increased risk of hypotension, AKI, hyperkalemia, angioedema
Direct Renin Inhibitors (DRI)	RAAS down-regulation by PRA reduction and inhibition of Angiotensinogen conversion to Ang I	Treatment of hypertension	24 h	No	↑ Renin concentration ↓ Renin activity	Dizziness, headache, muscle or joint aches, hyperkalemia, AKI
Angiotensin 1 receptor blockers (ARBs)	Blockage of Angiotensin II binding to its receptor (AT1R)	Treatment of hypertension, heart failure, chronic non-diabetic and diabetic renal disease	6–24 h	No	↑ Renin concentration ↔ Renin activity	Increased risk of hypotension, AKI, hyperkalemia

ACEi: angiotensin-converting enzyme inhibitors; RAAS: renin–angiotensin–aldosterone system; ACE: angiotensin-converting enzyme; AKI: acute kidney injury; DRI: direct renin inhibitors; PRA: plasma renin activity; Ang I: angiotensin I; ARBs: angiotensin receptor blockers; AT1R: angiotensin type 1 receptor.

A description of studies selected for our literature review is reported below and relative study design and results are resumed in Table 2. Adamczak et al. in subtotal nephrectomized rats with evidence of renal damage at biopsy evaluated both lab and histological renal outcomes in response to ACE inhibition. At baseline, after a 7-d period of adaption, as expected, blood pressure and albuminuria were significantly higher in animals with subtotal nephrectomy compared with sham operated rats. In addition, glomerulosclerosis and tubule-interstitial and vascular damage were more evident in sub-nephrectomized rats. Different length cycles of high-dose enalapril were conducted and sub-nephrectomized rats showed in all cases a significant reduction in albuminuria levels. Moreover, sub-nephrectomized rats, compared with sham operated groups, have a significant reversal of glomerulosclerosis and tubulointerstitial lesions [50].

**Table 2.** Summary of all studies included in the systematic literature review.

References	Study design	Study populations	Groups	Measurements	Baseline	After renal mass reduction	After anti-RAAS treatment	Adverse events (if any)
		*Animals*						
Adamczak et al. [50]	Randomized	58 Male -Sprague Dawley rats	•11 STN-X + ACEi •11 Sham	BP, UAER	*In Sham* *Cr* = 0.49 ± 0.006 mg/dl	*In STN-X* *Cr* = 0.92 ± 0.008 mg/dl	*In STN-X + ACEi* •*BP* = 116 mmHg •*UAER* = 4.85 ± 3.40 mg/24 h *In Sham* •*BP* = 127 mmHg •*UAER* = 0.47 ± 0.37 mg/24 h	None
Kelly et al. [51]	Randomized	80 Sprague-Dawley rats	•60 STN-X + ACEi •20 Sham	SBP, Prot, CCr	*In Sham* •*SBP* = 135 ± 10 mmHg •*Prot* = 1.3 ± 0.12 mg/d •*CCr* = 2.7 ± 0.14 ml/min	*In STN-X* *↑SBP=* (193 ± 11 mmHg) *↑Prot=* (6.24 ± 0.94 mg/d) *↓CCr=* (0.60 ± 0.11 ml/min)	*STN-X + ACEi for 12 weeks:* *↓SBP=*(131 ± 8 mmHg) *↓Prot=*(2.5 ± 0.43 mg/d) *↑CCr=*(1.23 ± 0.16 ml/min)	None
Mishina et al. [52]	Randomized controlled	6 Mongrel dogs	7/8 renal ablation in all animals	Cr, CCr, SBP,DBP, RAAS components	•*Cr* = 0.9 ± 0.2 mg/dl •*CCr* = 2.4 ± 0.5 ml/min/kg •*SBP* = 120.4 ± 10.3 mmHg •*DBP* = 71.3 ± 2.7 mmHg •*PRA* = 1.2 ± 0.9 ng/ml/h •*ANGI* = 474.4 ± 276.4 pg/ml •*ANGII* = 184.0 ± 143.2 pg/ml •*ALD* = 5.2 ± 6.5 ng/dl	*↑Cr* = 2.8 ± 0.5 mg/dl (*p* < .05) *↓CCr* = 1.0 ± 0.2 ml/min/kg (*p* < .05) *↑SBP* = 152.5 ± 18.9 mmHg (*p* < .05) *↑DBP* = 93.6 ± 11.7 mmHg (*p* < .05) *↑PRA* = 6.0 ± 1.4 ng/ml/h *↑ANGI* = 7312.8 ± 552.9 pg/ml *↑ANGII* = 3612.4 ± 1067.3 pg/ml *↑ALD* = 230.2 ± 84.5 ng/dl	*STN-X + ACEi for 2 weeks:* *↓Cr* = 2.5 ± 1.1 mg/dl *↑CCr* = 1.46 ± 0.14 ml/min/Kg *↓SBP* = 137.8 ± 12.4 mmHg *↓DBP* = 82.9 ± 3.6 mmHg (*p* < .05) *↑PRA* = 8.1 ± 1.3 ng/ml/h *↑ANGI* = 7980.8 ± 2521.6 pg/ml *↓ANGII* = 111.2 ± 106.2 pg/ml *↓ALD* = 13.4 ± 10.8 ng/dl	None
Singh et al. [53]	Randomized controlled	10 pure- bred Australian merino female sheep	•5 fetal Uni-X + ARBs •5 Sham + ARB	GFR, RBF, U_Na_V, FE_Na_	*In Sham* •*GFR* = 1.02 ± 0.1 ml/min/gkw •*RBF* = 10.1 ± 0.8 ml/min/gkw •*U_*Na*_V* = 2.0 ± 0.5 µmol/min/gkw •*FE_*Na*_ %*=1.4 ± 0.4	*↓GFR=*(0.62 ± 0.1 ml/min/gkw) [*p* < .001] *↓RBF = (*5.3 ± 0.5 ml/min/gkw) [*p* < .001) *↓U_*Na*_V = (*1.2 ± 0.2 µmol/min/gkw) *↓FE_*Na*_% =*(1.2 ± 0.4)	*ARB for 3 weeks in both groups:* *↑GFR=* most in Sham than Uni-X *↑RBF=* most in Sham than Uni-X *↑U_*Na*_V=* most in Sham than Uni-X **↑FE*_*Na*_*=**most in Sham than Uni-X	None
Singh et al. [21]	Randomized controlled	12 Australian merino male sheep	•6 fetal Uni-X + ARB •6 Sham + ARB	GFR, RBF, U_Na_V, FE_Na_	*In Sham* •*GFR* = 1.0 ml/min/gkw •*RBF* = 6 ml/min/gkw •*U_*Na*_V* = 1.5 µmol/min/gkw •*FE_*Na*_*=1.2%	*↓GFR=* (0.8 ml/min/gkw) [*p* < .001] *↓RBF=* (4.3 ml/min/gkw) [*p* < .001] *↓U_*Na*_V=* (0.8 µmol/min/gkw) (*p* < .01) *↓FE_*Na*_=*0.8%	*ARB for 3 weeks in both groups* *↑GFR=*most in Sham than Uni-X *↑RBF=* most in Sham than Uni-X *↑U_*Na*_V=* most in Sham (*p*=.003) than Uni-X *↑FE_*Na*_=*most in Uni-X than Sham	None
		*Humans*						
Wühl et al. [54]	Multicentric randomized controlled trial	385 children with CKD	71 pts with hypo/dysplasia randomized to receiving ACEi	GFR, 24 h BP, Prot	•*GFR* = 45.9 ml/min/1.73 m^2^ •*24 h BP* = 89.5 mmHg •*Prot* = 800 mg/m^2^/d	•*24 h BP=*118.3 ± 14.3 mmHg •*Prot=* 0.82 g	*After 6 months of ACEi* *↓24 h BP =* 109.4 ± 14.4 mmHg *↓Prot=* 0.36 g Progression in GFR reduction is delayed by use of ACEi.	*Hyperkalemia* (18 pts) *Hypotension* (2 pts)
Basturk et al. [8]	Retrospective	31 patients with SFK	21 of them receiving ACEi	GFR, Prot	*Prot=* 470 ± 662 mg/d	*Pts without ACEi* •*Last GFR* = 44.5 ± 33.4 ml/min •*Last Prot* = 0.95 ± 0.98 g/24 h	*Pts with ACEi* •*Last GFR* = 55 ± 21.1 ml/min •*Last Prot* = 0.48 ± 0.79 g/24 h	*ESRD* (2 pts)
Peco-Antìc et al. [55]	Prospective trial	14 children with CRM	5 children with CRM	Prot, GFR, 24 h BP	•*Prot=*0.93 ± 0.16 mg/mg •*GFR=*48.3 ± 13.4 ml/min/1.73 m^2^ •*24 h BP* = 85.4 ± 14.8 mmHg	*Before ACEi administration* •*Prot=*0.92 ± 0.18 mg/mg •*GFR=*45.8 ± 10.1 ml/min/1.73 m^2^ •*24 h BP=*90.4 ± 14.3 mmHg	*After ACEi administration* •*Prot=*0.23 ± 0.12 •*GFR* = 48.1 ± 1.8 ml/min/1.73 m^2^ •*24 h BP* = 80.5 ± 14.3 mmHg	Not reported
Nyame et al. [56]	Observational	900 patients underwent laparoscopic nephrectomy	338 patients treated with ARBs 562 no ARBs	GFR, Severe renal function, MACE	*Mean pre-operative GFR without RAAS blockade*: 87.2 ml/min *Mean pre-operative GFR with RAAS blockade:* 81.5 ml/min	*Mean post-operative GFR without RAAS blockade* (*day* 3)*:* 77.2 ml/min *Mean post-operative GFR with RAAS blockade* (*day* 3)*:* 69.3.5 ml/min	•*GFR* in ARBs patients: 69.1 ml/min •*GFR* in untreated patients:75.9 ml/min •*Rate of stage IV/V CKD in ARBs* patients in postoperative follow-up: 3.6% •*Rate of stage IV/V CKD in untreated patients* in post-operative follow-up: 4.4% •*Rate of MACE in continued ARBs* patients: 13.7% •*Rate of MACE* in discharged patients: 17.7%	Not reported
Hiremath et al. [57]	Systematic review of 21 randomized trials	1549 patients	ARBs/ACEi groups Controls	GFR, Prot	*Mean GFR=*61.6 ± 16.6 ml/min	*Mean GFR=*61.6 ± 16.6 ml/min	*GFR in ACEi/ARBs patients* was lower than in controls (−5.7 ml/min; 95% CI −8.7 to −2.8, *p* < .001) *In ACEi/ARBs patients was lower* than in controls (−0.47 g/d; 95% CI −0.86 to 0.08, *p* = .16)	*Anemia and hyperkalemia* (reported in 8 trials)

STN-X: subtotal nephrectomy; UAER: urinary albumin excretion rate; Uni-X: unilateral-nephrectomy; Sham: Sham operated animals; ARB: angiotensin receptor blocker; ACEi: angiotensin converting enzyme; SBP: systolic blood pressure; DBP: diastolic blood pressure; Cr: creatinine; CCr: creatinine clearance; ESRD : end stage renal disease; PRA: plasma renin activity; ANG I: angiotensin I; ANG II: angiotensin II; ALD: aldosterone; GFR: glomerular filtration rate; RBF: renal blood flow; FF: filtration fraction; U_Na_V: urinary sodium excretion; FE_Na_: fractional excretion of sodium; Pts: patients; CKD: chronic kidney disease; 24 h BP: 24 h blood pressure; N.P. : not performed; SFK : single functioning kidney; CRM: chronic renal malformation; MACE: major cardiovascular events; CI : confidence interval.

Kelly et al. tested the use of perindopril and a TGF-β inhibitor (tranilast) in nephrectomized rats that developed hypertension, and proteinuria associated with GFR decline and compared the results with a control group [51]. Perindopril, as well as, tranilast, showed significant nephroprotective effects, however, their association was found to be more effective in reducing blood pressure and proteinuria and was related with a milder decline of renal function. It is known that TGF-β is involved in the progression of renal damage due to its enhanced profibrotic and pro-apoptotic activity [51]. In addition, TGF-β levels correlate with RAAS up-regulation and are lowered by the use of anti-RAAS agents in patients with proteinuria. A synergic activity between perindopril and tranilast has to be supposed, being tranilast an enhancer of the pleiotropic activity of perindopril. At support, the study demonstrated that this combination therapy resulted in an incremental attenuation of glomerulosclerosis and tubule-interstitial fibrosis in association with evidence for a reduced activation of the TGF-β signaling pathway.

Mishina et al. practiced surgical 7/8 renal ablation in dogs and evaluated the potential causal role of the RAAS system in hypertension and chronic renal damage development. Accordingly to the hypothesis, 1 month after surgery, blood urea nitrogen, creatinine, blood pressure, plasma renin activity, ANG1–2 and aldosterone levels increased, while creatinine clearance decreased progressively. Benazepril (an ACE-inhibitor) was administered for two weeks in absence of relevant side effects. After treatment, renal dysfunction parameters were found to be significantly attenuated, while creatinine clearance increased. Despite the study was conducted in animals, ACE-inhibition showed a strong effectiveness in preventing the progression of the renal damage after the acute subtotal loss of one kidney [52].

As previously described, Singh et al. reported that fetal nephrectomy at 100 d of gestation in sheep causes a nephrogenesis compensation that leads to a 30%, rather than 50%, increase in nephron number at 6 months after birth. To understand whether RAAS inhibition could have positive effects on renal function in young nephrectomized sheep, losartan, an angiotensin receptor blocker (ARB), was administered. At baseline, compared to controls, nephrectomized animals had reduced GFR and RBF that significantly improved after 3 weeks of treatment. This suggests that the early administration of an anti-RAAS agent in subjects with congenital solitary kidney may prevent, during the after-born life, the progression of renal damage induced by a maladaptive response [21].

In a 5 years old ovine female of fetal unilateral nephrectomy, Singh et al. found a reduced RBF and GFR, as well as, an increase in ratios of intra-renal ANG1–7/ANG2 and ANG2 receptor/ANG1 receptor (AT2R/AT1R). On this basis, an infusion of ANG2 was tested in the presence of losartan and an increase in RBF and GFR was observed. However, deregulated ratios have been observed only in females, in which it may be supposed that the use of ARBs is more nephroprotective, while ACE inhibition may be pejorative. However, further investigations are needed [53].

Only few studies tested anti-RAAS agents in patients with solitary kidney with the aim to explore the impact of RAAS inhibition on renal function and chronic renal failure risk factors. Wühl et al. evaluated renal outcomes in a cohort of children with renal hypo/dysplasia treated with anti-RAAS agents. After 6 months of treatment, a significant reduction of 24 h blood pressure and proteinuria was observed. Even the progression in GFR reduction was delayed, although few adverse events were reported in 28% of children (18 patients developed iperkalaemia and two children had hypotension) [54]. Although anti-RAAS drugs appear to be nephroprotective in older children, ANG inhibition might induce pejorative effects in newborns also due to the role of ANG2 in kidney development and maturation [35,44].

In a retrospective study by Basturk et al. aimed at comparing renal outcomes in patients with congenital or acquired solitary kidney, an indirect observation on the impact of anti-RAAS drugs on renal function could be obtained. Data were limited to small sub-groups of patients and were found not clearly orientated for a beneficial effect of RAAS inhibition. However, authors acknowledged that further focused investigations were needed to elucidate this crucial issue [8].

Also Peco-Antić et al. conducted a prospective study by testing the ACE-inhibitor Ramipril in children with congenital renal malformations complicated with hypertension, proteinuria and chronic renal failure. In 36 months of follow-up, renal outcomes were found improved at each evaluation time-point: GFR decline resulted slowed in presence of a significant blood pressure and proteinuria reduction [55].

Safety of post-operative use of ARBs was evaluated by Nyame et al. in 338 out of 900 patients who underwent robot-assisted laparoscopic partial nephrectomy. Treatment group, compared with controls, showed a decreased risk to develop severe cardiac and renal function impairment both in the short and long term. This evidence is suggestive for the association of a satisfactory safety with known cardio-renal protective effects of ARBs in patients with renal mass loss [56].

An important issue is that of use of anti-RAAS agent in the post-transplant. Twenty-one randomized controlled trials were included in a systematic review focused on this topic by Hiremath et al. Results were substantially omogeneous and favorable to anti-RAAS use in kidney transplant recipients. Changes in glomerular filtration rate in response to ACEi/ARBs use were significantly lower in treated groups compared with controls and antiproteinuric effect was more evident in the long term [56].

## Conclusions

CKD is a common complication in patients having a reduced renal mass. A possible positive impact of anti-RAAS agents on GFR decline in patients with congenital or acquired solitary kidney has been partially explored so far. In this systematic literature review, we found only a limited quantity of data on this topic, nevertheless, suggesting that anti-RAAS agents might exert their nephroprotective effect also in solitary kidney patients, with limited evidence for serious adverse events. In children, in which ANG2 levels are crucial for sustaining a complete kidney development and maturation, the use of ARBs might be more appropriate. In women, based on the hypothesis of a hormonal interference on ANG2 levels and ATR1, the use of ARB agents might be more suitable. Indubitably, patients with solitary kidney complicated with hypertension and proteinuria deserve a pharmacological interference on RAAS system. Moreover, long-term nephroprotective effects have also been observed in kidney transplant recipients.

Based on the relevance of the topic, to affirm our conclusions and for a reliable assessment of the safety of anti-RAAS drugs in solitary kidney patients, a more extensive experimentation, preferably in clinical trials is strongly desirable.
